# Urethrovaginal Septum: A Histological Study on a Cadaver and Its Clinical Significance

**DOI:** 10.7759/cureus.73078

**Published:** 2024-11-05

**Authors:** Finn G Rieker, Mathangi Rajaram-Gilkes, Kelly Barr, Catherine Falkenstein, Kristi Fung, Daniel Marcincavage, Taylor Moglia, Ritika Segireddy, Renee Frank

**Affiliations:** 1 Medical Education, Geisinger Commonwealth School of Medicine, Scranton, USA; 2 Anatomic and Clinical Pathology, Geisinger Community Medical Center, Scranton, USA

**Keywords:** anterior vaginal wall, cadaveric study, pelvic organ prolapse, posterior urethral wall, primary vaginal tumor, stress urinary incontinence, urethral stricture, urethrovaginal fistula, urethrovaginal septum, vaginal orgasm

## Abstract

The academic literature concerning the tissue bridging the anterior vaginal wall and the posterior urethral wall, the urethrovaginal septum, is lacking and inconsistent, not only when compared to non-reproductive anatomy but also when compared to male reproductive anatomy. This knowledge gap must be addressed, given the implication of this tissue in numerous female reproductive pathologies and functions, including pelvic organ prolapse, intercourse and orgasm, urethral strictures, vaginal cancers, and stress urinary incontinence. This study seeks to characterize the histological composition of the urethrovaginal septum, including type I and III collagen proportions, elastin content, neurovascular distribution, and smooth muscle arrangement. Specimens were resected bilaterally from the bisected pelvic region of a postmenopausal female cadaver, spanning antero-posteriorly from the lumen of the urethra to the lumen of the vagina. The specimens were divided into medial and lateral portions, sectioned, and stained with hematoxylin and eosin, trichrome, reticulin, and elastin stains. Images were obtained through virtual microscopy. Type I collagen was confirmed as the major connective tissue component. Reticular fibers were mainly limited to blood and lymphatic vessel walls, as well as the perineurium and epineurium of nerve fibers. The elastic fiber content was minimal, appearing mainly in the lamina propria and vascular walls and interspersed with type I collagen in the connective tissue matrix of the septum. Smooth muscle bundles were mainly observed in the muscularis layers of both walls, but to a greater extent in the anterior vagina. These findings largely confirm those of the few published histological studies of this tissue while contributing to the current state of knowledge regarding the distribution of elastin and reticulin. Further research in premenopausal females and increased awareness of the microanatomy of the urethrovaginal septum is advised.

## Introduction

The academic literature is inconsistent with respect to the naming of the tissue between the posterior urethra wall and anterior vaginal wall, with some sources referring to it as the urethrovaginal septum (UVS), while others classify it as part of the deep perineal fascia or retropubic shadow, with additional discrepancies concerning the extent of this tissue [1-3]. This article will henceforth refer to the supporting structures between the posterior urethral and anterior vaginal walls as the UVS. Siccardi and Valle describe this septum as a supportive fascia posterior to the bladder that forms the anterior vaginal wall and closes the anterior hiatus left by the pubovisceral and puborectal bundles of the levator ani muscle [2]. Additional supporting structures in this region that have been identified include the pubovesical muscle, pubovesical ligament, lateral vesical ligament, and tendinous arch of the levator ani [4]. Another structure in this region is the pubocervical fascia, which covers the anterior aspect of the vagina and supports the female pelvic viscera [5]. It is located laterally to both the urethra and urinary bladder and attaches primarily to the ventral half of the arcus tendineus fascia pelvis, as its dorsal attachment is much weaker [6]. 

Varying reports of the collagen content and subtypes of the anterior vaginal wall exist, and the methods for the detection thereof are unstandardized [7]. Collagen forms supramolecular assemblies in the extracellular matrix and plays a distinct role in tissue shape and organization, as well as the regulation of cell proliferation, differentiation, and migration [8]. The superior portion of the UVS is composed of loose connective tissue, whereas the inferior portion is composed of dense connective tissue and is not well-separated from the anterior vaginal wall [4]. A 2021 analysis of high-resolution sections by Li et al. revealed that the superior and inferior portions of the UVS were on average 18.3 mm and 13 mm in length, respectively [4]. A 2017 histological analysis of the posterior urethra and vaginal wall by Mazloomdoost et al. revealed the following layers and corresponding thicknesses: vaginal epithelium (0.09 mm), lamina propria (0.90 mm), fibromuscular layer (2.36 mm), vaginal adventitia (0.84 mm), and posterior urethral wall thickness (5.21 mm), consisting of the urethral muscularis, urethral lamina propria, and urethral epithelium [9]. The same analysis also identified a layer of fibroconnective and adipose tissue separating the anterior vagina and urethra, the UVS, on 41% of slides and that the vascularity, layer thickness, and innervation were consistent along the entire length of the vaginal wall [9]. Another histologic study of the area between the bladder, urethra, and anterior vagina by Hamner et al. showed dense fibrous tissue, smooth muscle bundles, scant adipose tissue, blood vessels, and nerves, but no evidence of a discrete fascial layer or pubourethral ligaments [10].

Nevertheless, despite substantial progress in gross and microscopic mapping of human anatomy, there has been relatively minimal literature on the histologic characterization of the UVS and surrounding structures. A 2021 systematic review by Roch et al. revealed that from 1971 to 2021, only three studies specifically investigated the gross anatomy either with or without histological analysis of the pubocervical fascia and immediately adjacent structures [11]. The lack of literature on the supporting structures of the female urethra is due in part to the difficulty associated with the dissection of this area [12], while studies employing MRI or CT provide only limited articulation and resolution of structures [4]. Additionally, social factors, like those demonstrated by the deletion of the clitoris label from early Grey's Anatomy versions, have culminated in a limited understanding of female anatomy including sexual function and dysfunction, when compared to male reproductive anatomy [13]. A 2022 analysis of PubMed and grant databases by Mercuri and Cox also revealed that the number of research articles published and research grant funding awarded was significantly lower for projects exploring reproductive organs compared to those of non-reproductive organs [14]. 

This article characterizes the components of the UVS in a female cadaveric specimen utilizing multiple histological staining preparations in an effort to bolster the limited existing literature and better describe the tissue that bridges the urethra and vagina while applying the findings to relevant clinical correlates.

## Materials and methods

**Figure 1:**
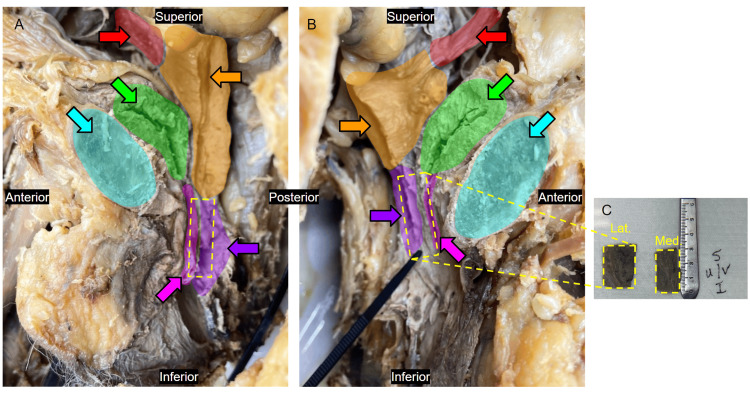
The image indicates the site of the tissue section. Bisected (A) right and (B) left hemipelvic regions of a female cadaver with highlighted genitourinary structures alongside (C) the medial and lateral halves of a resected specimen. Urethra (pink), vagina (purple), bladder (green), uterus body (orange), fallopian tubes (red), and pubic symphysis (blue).

This study utilized a donated elderly female cadaver at Geisinger Commonwealth School of Medicine at the end of the reproduction block. This cadaver was assigned for the normal dissection process during the learning activity of the gross anatomy of the reproductive system. The pelvic cavity was normal with all reproductive organs intact and with no evidence of surgical procedures. The request for this study was submitted to the institutional review board, which was waived as the details of the individual were de-identified. The cadaver was bisected along the sagittal plane from the perineal region to the lumbar region, exposing the genitourinary region as left and right halves for tissue access (Figure 1A, 1B) as a normal procedure to view and identify the female reproductive organs and the peritoneal reflections as a part of a routine anatomy lab. Figure 1A shows a gross image of the right hemisected pelvic region with the pubic bone anteriorly and the bladder posteriorly followed by the uterus body and their respective passages highlighted. Figure 1B shows the gross image of the left hemisected pelvic region with the pubic bone anteriorly followed by the bladder and uterus posteriorly also highlighted. Following bisection, 3 cm × 1 cm × 1 cm pelvic specimens were resected bilaterally using a No. 11 scalpel, spanning antero-posteriorly from the lumen of the urethra to the lumen of the vagina, thereby encompassing the posterior urethral wall, the UVS, and the anterior vaginal wall (Figure 1C). These specimens were further divided into medial and lateral portions and transported to the lab in formalin (Figure 1C).

The specimens were embedded within paraffin blocks, sectioned into 15 µ sections using a microtome, and stained with hematoxylin and eosin (H&E), Mallory's trichrome, reticulin, and elastin stains. These stains were chosen to identify type I collagen (H&E), reticular fibers (reticulin), and elastic fibers (elastin) and to differentiate between type I collagen and smooth muscle (Mallory's trichrome) and elastic fibers. Images were obtained through virtual microscopy and are discussed below.

## Results

The sections presented herein show the vaginal mucosa, composed of the stratified squamous non-keratinized epithelium, and the lamina propria; the submucosa, containing the connective tissue and large blood vessels; the muscularis, containing smooth muscle bundles in the superior two-thirds and the skeletal muscle in the inferior third; and the outermost adventitia, containing the loose connective tissue. The connective tissue in the region where the urethral and vaginal walls were observed merging was identified using the aforementioned stains.

**Figure 2:**
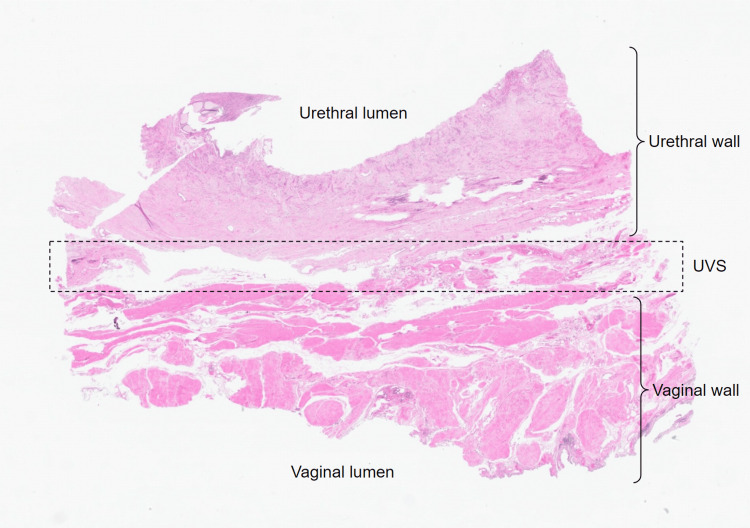
Low-magnification virtual microscopy image of a section from the medial aspect of the left-sided specimen stained with H&E. UVS: urethrovaginal septum; H&E: hematoxylin and eosin

The section taken from the medial aspect of the left-sided specimen involves the walls of the urethra and vagina adjacent to one another (Figure 2). Although the urethral and vaginal epithelia were patchy due to the nature of the specimen collection, they served as landmarks for identifying the luminal surfaces. Just above the vaginal epithelium on this slide is the lamina propria, traversed by large blood vessels (Figure 2). The muscularis located just above this layer contained bundles of smooth muscles, followed by the adventitia of the vaginal wall, which was observed to be shared back-to-back, between the walls of the urethra and vagina (Figure 2). In this UVS region, H&E staining revealed microanatomical structure and enhanced the appearance of type I collagen, enabling the visualization of dense irregular connective tissue, which was composed mainly of type I collagen, as well as blood vessels and lymphatics (Figure 2). H&E staining also revealed type I collagen in the lamina propria, smooth muscles in the muscularis, and type I collagen in the adventitia, which appeared to extend into the UVS region from the adventitia of both walls (Figure 2). Small pockets of adipose tissue were observed scattered throughout the UVS.

**Figure 3:**
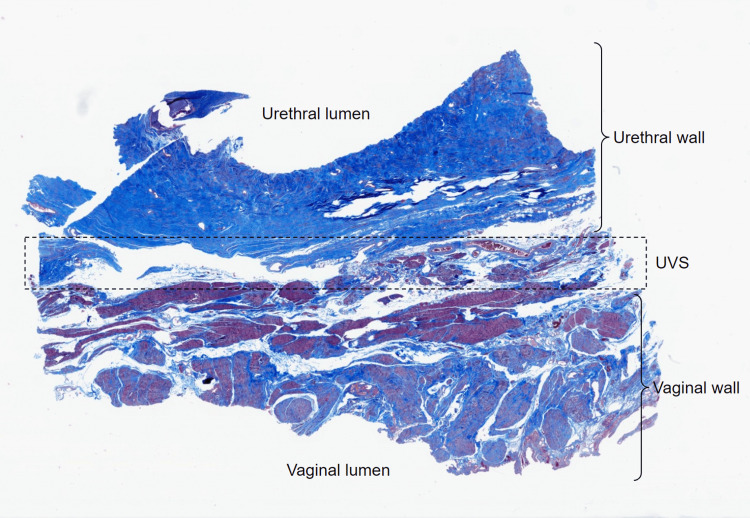
Low-magnification virtual microscopy image of a section from the medial aspect of the left-sided specimen stained with Mallory's trichrome. UVS: urethrovaginal septum

Mallory's trichrome staining clearly differentiated type I collagen (blue) from smooth muscle (brown) (Figure 3). Notably, smooth muscle distribution was significantly denser in the anterior vaginal wall than the posterior urethral wall. This could be because the specimens were predominantly harvested from the superior two-thirds of the UVS, while the muscle content of the urethra was found to be mainly in the inferior third. Trichrome staining substantiated the abundant presence of type I collagen within the UVS, alongside scattered blood vessels and lymphatics (Figure 3).

**Figure 4:**
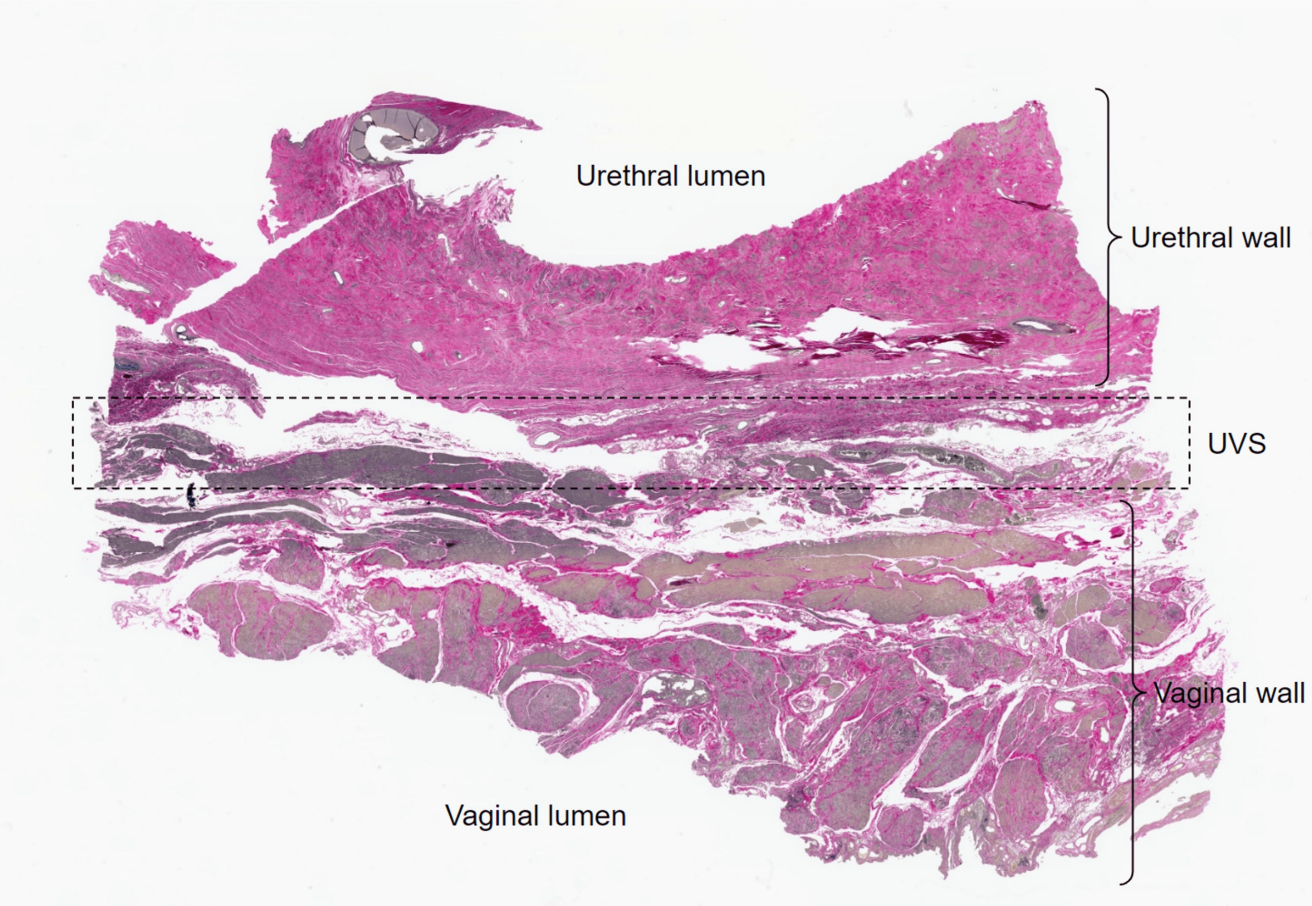
Low-magnification virtual microscopy image of a section from the medial aspect of the left-sided specimen stained with an elastin stain. UVS: urethrovaginal septum

Elastin staining revealed that elastic fibers are present within the UVS, though not in abundance. The elastic fibers were found mainly in the lamina propria, as a component of vascular walls in the vessels of adventitia, and intermingled with type I collagen within the connective tissue matrix of UVS, though again not in abundance (Figure 4).

**Figure 5:**
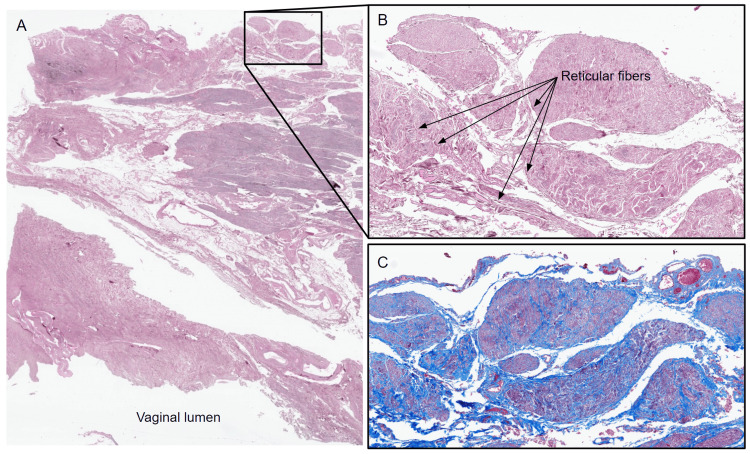
Low-magnification virtual microscopy images of sections stained with a reticulin stain. Low-magnification virtual microscopy images of sections from the lateral aspect of the right-sided specimen stained with a reticulin stain (A). (B) 10× magnification of a UVS region from the reticulin-stained section in panel (A) showing reticular fibers. (C) 10× magnification of the same UVS region in panel (B) stained with Mallory's trichrome, showing neurovascular bundles. UVS: urethrovaginal septum

Examination of reticulin-stained slides via virtual microscopy revealed reticulin fibers to be a component of blood vessel walls and part of the perineurium and epineurium of nerve fibers in the urethra-vaginal septal regions (Figure 5A). This was best observed at high magnification (Figure 5B). Overall, reticular fibers were found not to be a predominant component of the connective tissue in this region. The abundant nerve fibers in the septum were best visualized via Mallory's trichrome staining (Figure 5C).

**Figure 6:**
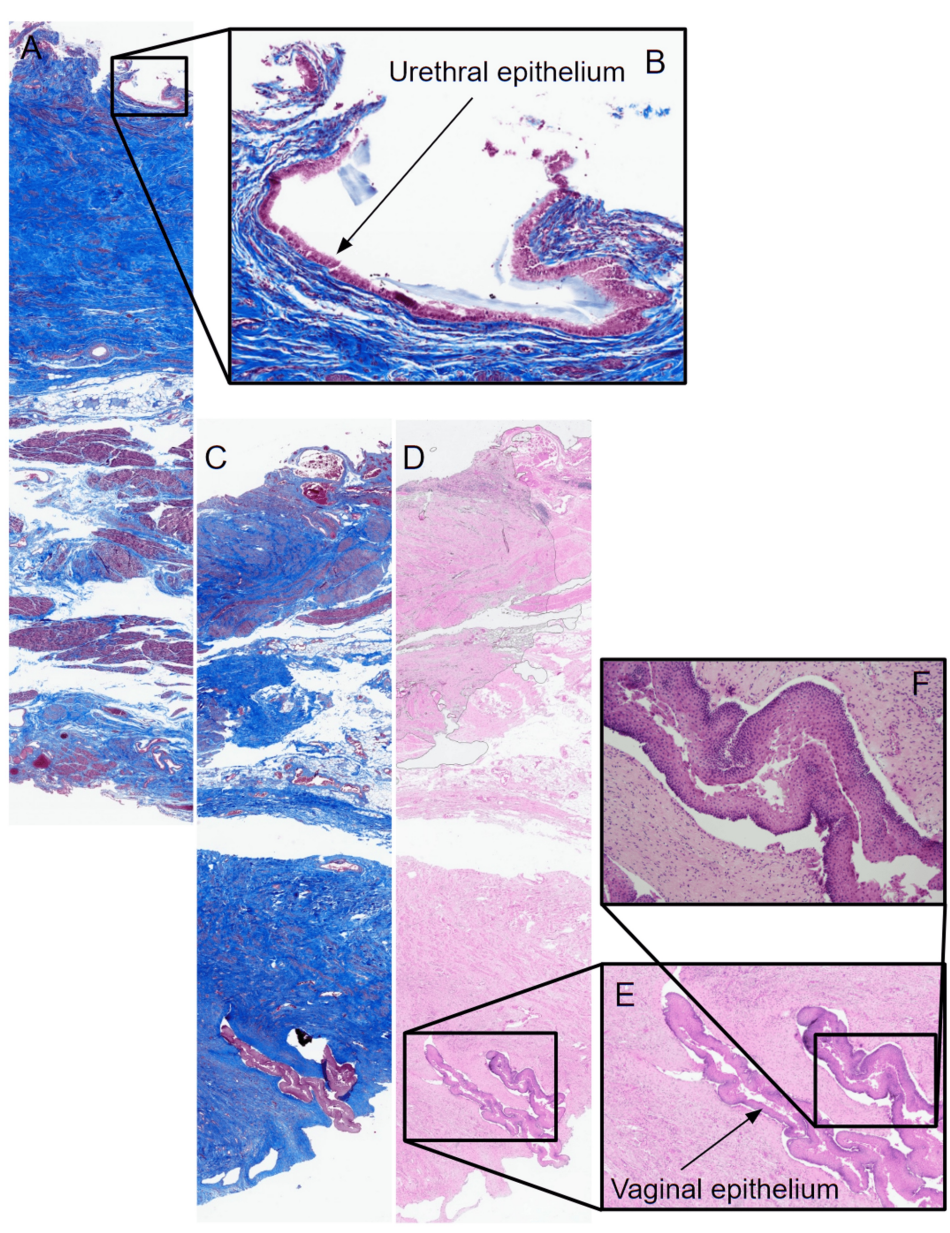
Images to highlight the epithelia using trichrome and H&E stains. Virtual microscopy images from (A-B) the lateral aspect of the left-sided specimen and (C-F) the lateral aspect of the right-sided specimen, stained with Mallory's trichrome (A-C) and H&E (D-F) stains, showing the urethral epithelium, urethral wall, UVS, vaginal wall, and vaginal epithelium. (B) 20× magnification of the urethral epithelium from panel (A). (E) High and (F) higher (10×) magnifications of the vaginal epithelium from panels (C-D). H&E: hematoxylin and eosin; UVS: urethrovaginal septum

Figure 6 is a compilation of virtual microscopy images of sections from the left- and right-sided specimens to produce a visual representation of the entire span of the urethral and vaginal walls, including their respective epithelia and the UVS. High magnification allows for the visualization of both epithelia. The pseudostratified columnar urethral epithelium is clearly visible here, immediately lining the urethral lumen, followed by the lamina propria which is made of loose connective tissue (Figure 6B). The stratified squamous non-keratinized vaginal epithelium is also clearly identifiable at this magnification (Figure 6E-6F).

## Discussion

Through extensive staining of multiple sections of the posterior urethral wall and anterior vaginal wall, this study confirmed that type I collagen is the major connective tissue component, extending from the incompletely separated urethral and vaginal adventitias into the shared UVS. Investigating the content and distribution of reticular fibers, which are composed of type III collagen, within the UVS was a central aim of this study. Short, thin, branching reticular fibers were mainly limited to the walls of blood and lymphatic vessels, as well as the perineurium and epineurium of nerve fibers. Although nerve fibers were abundant, reticular fibers were not a predominant component of the UVS overall. The elastic fiber content was minimal, appearing mainly in the lamina propria and vascular walls and interspersed with type I collagen in the connective tissue matrix. Smooth muscle bundles were mainly observed in the muscularis layers of both the urethral and vaginal walls and to a greater extent in the anterior vaginal wall. Minimal adipose tissue was also observed throughout the UVS. These findings largely confirm those of the few published histological and microanatomical studies of the UVS while contributing to the current state of knowledge regarding the distribution of elastin and reticulin.

Investigators in previous MRI studies have analyzed the functional capacity of the urinary continence mechanism by integrating the urethra and relevant supporting structures into one consolidated unit [12]. However, an understanding of the precise anatomic definition of these structures is necessary for the formulation of proper diagnoses and successful surgical interventions [12]. Stretching, damage, and irregularities to the anchoring ligaments and structures present in this region have been implicated in pathologies, including uterine prolapse, congenital anomalies such as urethral-vaginal fistulas, and others [15,16]. Therefore, it is crucial to adequately characterize this region, including the UVS.

Increased intraabdominal pressure and pelvic organ prolapse (POP)

These histological findings contribute to the understanding of the role of the UVS in POP, which is related to states of increased intraabdominal pressure due to straining, such as labor and defecation. Currently, there is limited information on the histological changes within this region in women who develop POP, and most studies include patients in the postmenopausal state, which is complicated by changes due to aging [7]. Also in a postmenopausal female, this study found type I collagen to be the major collagen component in the UVS, while reticulin and elastin were minor components, which are hypothesized to provide support within the pelvic floor.

Excessive straining can weaken the musculature of the vaginal wall, as well as the pelvic floor muscles, and damage the pudendal nerve, thereby lowering the pelvic floor and increasing the size of the vaginal outlet [17]. The relatively muscular nature, abundant collagen fibers, yet scarce elastin found within the UVS of the cadaveric specimen support the explanation of stretching causing weakness over time. An example of straining that can change vaginal wall integrity is defecation. Excessive stress during defecation can cause stretching of the anterior vaginal wall which can lead to anterior vaginal wall prolapse and thus POP [18]. Kose et al. show that squatting during defecation is associated with increased severity of symptoms for patients with anterior vaginal wall prolapse compared to patients who sit for defecation, due to the increased intraabdominal pressure that occurs when squatting compared to sitting [18].

Vaginal parity and aging also have significant effects on pelvic floor muscle architecture and vaginal smooth muscle. Post-childbirth vaginal tissue shares characteristics with prolapsed vaginal tissue, such as smooth muscle disorder and increased extracellular matrix, and 20% of postmenopausal females suffer from pelvic floor dysfunction [4,19]. The notable age-related changes manifest as a decreased physiological cross-sectional area across all pelvic floor muscles, but pelvic floor muscle mass did not significantly differ between parous and nulliparous specimens in the younger and older cohorts [20]. The smooth muscle mass of the vaginal wall decreases with age due to decreased estrogen levels [19]. While estrogen replacement therapy has been reported to alleviate the vaginal symptoms of age-related estrogen level decreases, the evidence is lacking and doesn't include data concerning smooth muscle [19]. Surgical mesh implantation is a common intervention, but the relatively high risk of known complications may be attributable to the knowledge gap concerning the contractile properties of the vagina, which helps explain the poor outcomes in the surgical treatment of POP [19]. This study revealed much denser smooth muscle content in the anterior vaginal wall than in the posterior urethral wall, bolstering the histological reference for vaginal wall smooth muscle content, the atrophy of which is implicated in vaginal prolapse.

Women with POP also exhibit increased elastin metabolism with greater expression of the elastin-degrading enzyme matrix metalloproteinase-9 (MMP-9) [21]. This increased metabolism is thought to be due to vaginal remodeling due to increased mechanical stretch [21]. The low levels of elastin found within the UVS of the postmenopausal female cadaver in this study support the role of elastic fibers within the UVS in age- and possibly childbirth-related vaginal prolapse and demonstrate the potential risks associated with elastic fiber disruption during interventional procedures.

Stress urinary incontinence (SUI)

The UVS is particularly relevant when treating patients with SUI, which is characterized by involuntary urination with increased abdominal pressure, such as either sneezing, coughing, jumping, or physical exertion, and impacts about 50% of females worldwide [22]. The causes of SUI are multifactorial and include risk factors such as increased age, damage or injury commonly associated with multiple vaginal deliveries, and genetic predispositions [23]. When treating individuals with SUI, the gold standard of treatment is the pubovaginal sling (PVS) [24]. Typically, a PVS is inserted at the bladder neck or mid-urethra such that the sling becomes incorporated into the endopelvic fascia through fibrosis [22,24]. Although the pelvic diaphragm depends on the levator ani musculature, both the pubocervical fascia and the ureteropelvic ligament provide additional support [22]. Slings provide resistance to the urethra during stress maneuvers and, therefore, prevent SUI [24]. Additional groups have shown to benefit from the use of non-surgical interventions, such as physical therapy to strengthen the surrounding musculature, in preparation for surgical intervention [23]. Furthermore, retrospective analyses have found that there are correlations between detrusor overactivity and UVS thinning; however, the literature is lacking, and thus, the relationship remains unclear [25]. Ultimately, surgical placement of a PVS requires familiarity with urethral surroundings, especially the UVS; the findings of investigations like this one, such as those concerning the neurovascular and collagen distributions throughout the UVS, should be applied in an effort to lower the complication rates of such surgeries.

Sexual function

Although the understanding of female sexual function is still relatively within its infancy, these cadaveric findings yield insight into the normal physiology of intercourse. Currently, there is still speculation of a functional correlation between the thickness of the UVS and the occurrence of a vaginal orgasm [26]. A 2008 study utilized introital ultrasonography to characterize the UVS thickness as a measure of anatomical variability among a cohort and found a direct, significant correlation showing that the distal, middle, and proximal segments of the UVS are thinner in women without vaginal orgasm [26]. Women with a thicker UVS were more likely to experience vaginal orgasm, with the highest correlation observed for a thicker distal segment [26]. Three-dimensional ultrasonographic reconstruction has demonstrated that the structures within the UVS are gland-like without rich vascularization and its volume is correlated with serum androgen concentrations and time since intercourse [27]. UVS contents like nervous and connective tissue, and their role in transmitting the effects of intercourse to the clitoris, make the UVS critical to the physiology of intercourse and again stress the risks of damaging this tissue during surgical intervention.

Urethral strictures

Urethral strictures cause narrowing of the urethra, secondary to inflammation, and fibrotic scar tissue formation due to mechanical or iatrogenic injury or idiopathic causes; this may lead to dysuria, a weakened stream, urinary frequency, urgency, and difficulty emptying [28]. In these cases, densely packed connective tissue fibers replace the normal urethral spongiosum via the increased expression of connective tissue growth factors [28-30]. Given the already-abundant type I collagen content in the UVS, as well as the urethral and vaginal walls, alteration of growth factor expression may disrupt the connective tissue balance in the region. Such an imbalance has been confirmed to be implicated in strictures of the urethral spongiosum, as normal spongiosum is composed of 75.1% type I collagen and 24.9% type III collagen, whereas urethral stricture scar tissue was composed of 83.9% type I collagen and 16.1% type III collagen [29]. These differences in collagen proportions explain the non-compliance of urethral stricture scar tissue and may offer insight into the potential implication of collagen imbalances in dysfunctions of vaginal compliance, especially with aging or damage.

Although female urethral strictures are rare, with only 10% of females experiencing obstructive voiding having a stricture, the symptoms nonetheless hinder the quality of life [31]. Currently, the first-line treatment for female urethral strictures is vaginal dilation; however, dilations only have a 47% composite success rate, and there are no guidelines on the surgical repair of female urethral strictures [31,32]. While male patients with short, thin strictures and minimal scar tissue also receive conservative, minimally invasive treatment, male patients with recurrent, thick, or long strictures and dense scar tissue usually undergo urethroplasty, which has higher cure rates than urethral dilation in females [33]. It is hypothesized that the rarity of the condition, the lack of experience among surgeons, and the risk of complications currently hinder female urethroplasty from becoming the norm, especially after dilation fails to improve quality of life [31]. Regardless of the surgical techniques used, which include anterior vaginal wall flap urethroplasty among others, postoperative homeostasis remains a challenge, necessitating vaginal packing [31]. No comparative trials have been conducted to determine the ideal approach or graft type, leaving the decision regarding surgical technique solely up to the surgeon's preference [31]. New information regarding the composition and function of surrounding tissue should influence the risk-benefit analyses of surgeons treating female urethral strictures moving forward, including findings like those of this study on the connective tissue distribution, vascularity, and nervous tissue within the UVS. Histological examination of graft sites may also mitigate the risk of complications like urethrovaginal fistulas and should be investigated in future studies.

Tumors and fistulas

Primary vaginal cancers are rare, with roughly 84% of vaginal cancers arising secondarily from the surrounding anatomy, such as the cervix and endometrium [3]. Tumors arising from the UVS are even rarer, but cases of masses without urethral or vaginal mucosa involvement have been reported [3]. Tumors in the UVS often exhibit vague symptomatology including urinary retention and recurrent urinary tract infections, which increases the risk of misdiagnoses in favor of more benign conditions such as urethral caruncles or prolapse [3]. For the identification of all potential diagnoses, but especially cancers within the UVS, a thorough examination including the palpation of the anterior vaginal wall should be performed on patients presenting with these symptoms [3].

Urethrovaginal fistulas can be congenital but are most commonly a result of prior gynecological surgery or prolonged obstructed labor and present with symptoms similar to tumors of the region [3]. Fistulas proximal to the urethral sphincter result in incontinence, while those distal may be asymptomatic or result in a splayed urinary stream [34]. Untreated fistulas increase the risk for the development of vaginal stones due to pathologic calcification and stagnant urine, which may progress to worsening obstructions and infections of the urinary tract [35]. Given the association between surgical intervention for other genitourinary conditions and the development of urethrovaginal fistulas, in addition to most fistulas requiring surgery themselves, the findings of this study and others like it are crucial for the optimization of women's healthcare.

Limitations

Despite contributing to the body of research on the histological composition of the UVS, this study does possess some limitations. For instance, the use of cadavers in histological studies introduces postmortem shrinkage of connective tissue. The practice of cadaveric dissection also presents certain limitations. Although microscopic examination of stained cadaveric sections provides higher resolution than imaging modalities, the process of dissection can introduce artifacts, such as the epithelial sloughing seen in this study. Furthermore, while the age at death enabled investigation of the age-related and postmenopausal changes associated with certain pelvic pathologies, it also limits the generalizability of the findings. The shortage of published research on the microanatomical composition of the UVS, as well as inconsistencies regarding its extent and naming, also limited the literature review stage of this study. Finally, the use of just one cadaver was a result of the discrepancy between cadavers allocated for educational and research purposes, but it nonetheless limited conclusions concerning the reliability of the findings.

## Conclusions

This histological study adds to the body of research on the tissue bridging the posterior urethral and anterior vaginal walls, the UVS, given the lack of information available in the existing literature regarding its potential implications in numerous female reproductive pathologies, functions, and surgical procedures. The connective tissue matrix of this septum consisted predominantly of type I collagen, with reticular fibers being concentrated in the perineurium and epineurium of nerves, and walls of blood vessels and lymphatics. Elastic fibers were abundant in the lamina propria and smooth muscles in the muscularis layers of both passages, but not in the septum. These results are useful given previous findings including, but not limited to, the collagen type imbalance in urethral stricture scar tissue, the increased rates of elastin metabolism and implication of vaginal smooth muscle in POP, as well as the overall relevance of the composition of the septum to the study of rare primary vaginal tumors. The profound vasculature and nerve tissue content of this septum is implicated in sexual functions and poses a risk of injury in surgical interventions for conditions such as urethral fistulas and SUI. Though this study involves an elderly cadaver, it contributes to the deficient pool of current literature on this subject, and we recommend further exploration of this region with larger sample sizes, in pre- and perimenopausal women.
